# Normal Gain with Corrective Saccades in the Video Head Impulse Test: Clinical Implications and Diagnostic Considerations

**DOI:** 10.3390/diagnostics16142195

**Published:** 2026-07-14

**Authors:** Goun Choe, Chang-Hee Kim, Dong-Han Lee

**Affiliations:** Department of Otorhinolaryngology-Head and Neck Surgery, Konkuk University Medical Center, Konkuk University School of Medicine, Research Institute of Medical Science, 120-1 Neungdong-ro (Hwayang-dong), Gwangjin-gu, Seoul 05030, Republic of Korea; gounchoemd@gmail.com (G.C.);

**Keywords:** vestibulo-ocular reflex, saccades, semicircular canals/physiopathology, head impulse test, vestibular diseases/diagnosis

## Abstract

**Background:** The video head impulse test (vHIT) is a standard tool for assessing semicircular canal function through vestibulo-ocular reflex (VOR) gain and corrective saccades (CS). Although reduced gain accompanied by CS typically indicates vestibular hypofunction, a paradoxical pattern—normal gain with CS—is occasionally observed, and its clinical significance remains unclear. **Methods:** We retrospectively analyzed 1573 patients who underwent both vHIT and bithermal caloric testing between September 2022 and September 2025. Among them, 1174 subjects with normal bilateral horizontal VOR gains (0.8–1.2) were evaluated for CS. CS were defined as at least three consistent refixation saccades on one side, verified visually. Subjects were categorized into Right-, Left-, and Bilateral-CS groups. Comparisons were made between CS(+) and CS(−) sides, as well as among groups, using paired and one-way ANOVA tests. **Results:** CS were identified in 46.3% (544 in 1174) of subjects with normal VOR gains, most frequently on the right side. The side showing CS exhibited significantly lower caloric responses than the contralateral side (*p* < 0.001). Bilateral CS were more common in older patients. Canal paresis values indicated that the CS side corresponded to the weaker caloric side, suggesting subtle unilateral hypofunction. **Conclusions:** Even with normal gain, the presence of CS may reflect mild vestibular asymmetry rather than a benign artifact. In addition, bilateral CS were associated with older age, suggesting a possible contribution of age-related vestibular or compensatory mechanisms. Careful control of methodological biases and integration with other vestibular tests are essential for interpretation.

## 1. Introduction

The video head impulse test (vHIT) evaluates semicircular canal function by quantifying the vestibulo-ocular reflex (VOR) during brief, examiner-delivered head rotations of small angular displacement. Effective gaze stabilization requires eye movements that are equal in velocity and opposite in direction to head motion; this relationship is expressed as the VOR gain, the ratio of eye velocity to head velocity [1]. In healthy vestibular function, this compensatory response is preserved, yielding a gain near unity without corrective saccades (CS). In unilateral vestibular hypofunction, insufficient eye velocity during impulses toward the affected side results in reduced gain and the emergence of CS, which may occur either after the head movement (overt) or during the impulse itself (covert).

Although covert saccades were initially characterized using scleral search coil techniques in experimental settings [2], high-speed video recording has enabled their reliable identification in routine clinical practice [3,4]. By providing a noninvasive, real-time, and canal-specific assessment of VOR function, vHIT has become an integral component of contemporary vestibular evaluation. In clinical practice, however, a subset of patients exhibits CS despite normal VOR gain, presenting a diagnostic paradox. This finding raises questions regarding potential mechanisms such as subtle canal dysfunction below the detection threshold, frequency-dependent impairment, central adaptation, or measurement artifacts. Previous studies, including Yang et al. [5], have reported CS even in healthy subjects—defined as occurring in more than two out of ten impulses without a velocity threshold—while later studies proposed stricter definitions (e.g., peak velocity >100°/s in >50% of impulses) [6]. Yet, no universally accepted standard for CS definition currently exists.

The clinical meaning of normal-gain with CS remains unclear, although emerging evidence suggests that CS with preserved gain may correspond to reduced caloric responses, implying mild or frequency-specific vestibular dysfunction [6]. Caloric testing primarily assesses horizontal semicircular canal and superior vestibular nerve function using low-frequency stimulation, whereas vHIT evaluates all six semicircular canals using physiological high-frequency head rotations. Because these tests examine vestibular function across different frequency ranges, they provide complementary information regarding peripheral vestibular function [7,8]. Data on its prevalence and diagnostic relevance in real-world dizzy patients, however, remain limited. This study aims to investigate the occurrence and clinical implications of normal-gain with CS using a large patient cohort who underwent both vHIT and caloric testing. By applying a conservative detection approach and a broad CS definition similar to Yang et al. [5], we sought to determine the prevalence, lateralization, and caloric correlates of this pattern, thereby clarifying its diagnostic significance and the proper interpretation in vestibular assessment.

## 2. Materials and Methods

### 2.1. Subjects

A total of 1573 patients who completed both video head impulse test (vHIT) and bithermal caloric test during vestibular function assessment at our hospital between September 2022 and September 2025 were retrospectively analyzed. No restrictions were imposed regarding the underlying vestibular diagnosis, and all patients who underwent both tests during the study period were initially eligible for inclusion. Among them, 1174 subjects (74.6%) showing normal horizontal canal VOR gains (>0.8 and <1.2 on both sides [9,10,11]) were selected for further analysis of CS. Vertical canal vHIT responses were not analyzed in the present study. This retrospective study was approved by the Institutional Review Board of Konkuk University Medical Center (KUMC IRB No. 2025-10-042; approved on 4 November 2025), and the need for informed consent was waived due to the retrospective analysis of de-identified clinical data and the minimal risk posed to participants. In most subjects, vHIT and caloric testing were performed on the same day as part of a routine vestibular function assessment, although a small number of patients underwent the two tests on separate but temporally proximate dates because of scheduling considerations.

### 2.2. Video Head Impulse Test (vHIT)

The video head impulse test was performed using a three-dimensional ICS Impulse vHIT system (GN Otometrics, Taastrup, Denmark). The lightweight goggles equipped with a high-speed video-oculography camera and inertial sensors were securely fitted to each subject’s head. Participants were seated 1 m away from a visual target and instructed to maintain gaze fixation throughout testing. Calibration was performed prior to testing to ensure accurate eye–head alignment. Head impulses were delivered unpredictably in both horizontal directions with small amplitude (angular displacement of 5–15°) and high peak velocity (150–200°/s) while subjects kept their gaze on the fixed target. At least 10–15 impulses were applied to each side. All vHIT examinations were performed by a single experienced examiner with more than one year of vHIT testing experience using a standardized testing protocol. Eye movements were recorded using the default right-eye tracking configuration of the ICS Impulse system, which was applied consistently throughout the study.

Eye and head velocities were recorded simultaneously, and the vestibulo-ocular reflex (VOR) gain was automatically calculated as the ratio of the area under the curve (AUC) for eye velocity to that for head velocity. CS were identified based on the presence of compensatory eye movements following the head impulse in the direction opposite to the rotation. Only CS elicited during horizontal head impulses were included in the present analysis.

### 2.3. Caloric Test

Before caloric testing, all subjects underwent routine otoscopic examination to assess the condition of the external auditory canal and tympanic membrane. The bithermal caloric test was performed using water at 30 °C and 44 °C for 40 s per irrigation while participants lay supine with the head elevated 30° from the horizontal plane. When water irrigation was contraindicated, such as in patients with chronic otitis media with tympanic membrane perforation or after ear surgery, air caloric testing (24 °C and 50 °C for 60 s) was conducted instead. Water and air caloric stimulation were delivered using the NCI-480 and AirCal caloric irrigators, respectively (ICS Medical, Schaumburg, IL, USA). Eye movements were recorded using a video nystagmography system (CHARTR VNG, ICS Medical, Schaumburg, IL, USA), which provides binocular eye movement recording at a sampling rate of 60 Hz, with a spatial resolution of 0.1° and a measurable range of ±30° in both the horizontal and vertical planes. The peak slow-phase velocity (SPV) was automatically calculated for each ear after each irrigation. Canal paresis (CP) was calculated using Jongkees’ formula.

### 2.4. Corrective Saccades Definition and Subject Grouping

The presence of CS was primarily determined using the automated detection provided by the vHIT system (ICS Impulse, GN Otometrics, Taastrup, Denmark). All detected traces were visually inspected by an experienced examiner to confirm their validity. A corrective saccade was considered present when at least three consistent refixation saccades were identified on the same side during head impulses. Because there is currently no consensus regarding the optimal definition of CS, we adopted a previously published criterion similar to that of Yang et al. [5] rather than proposing a new study-specific threshold. This approach was intended to facilitate comparison of our findings with those of previous studies. Saccades with low peak eye velocity (less than approximately half of the peak head velocity) or those considered artifacts based on irregular waveform morphology or inconsistent timing were excluded. Covert and overt CS were not separately analyzed, as the primary aim was to assess the presence or absence of CS rather than their latency characteristics. This simplified approach was adopted in the present study to provide an initial overview of the features in cases with normal VOR gain and CS, while acknowledging that more detailed subclassification could be informative in future analyses.

Participants with normal bilateral horizontal canal vHIT gains (0.8–1.2) were categorized according to the side showing CS. Those with CS observed only on the right side were classified as the Right-CS group, and those with CS only on the left side as the Left-CS group. Subjects who exhibited CS on both sides were defined as the Bilateral-CS group.

For analyses comparing the side with and without CS irrespective of laterality, subjects with unilateral CS (i.e., the Right-CS and Left-CS groups) were combined into a single unilateral-CS group. In this analysis, the side exhibiting CS was designated as the CS(+) side and the contralateral side without CS as the CS(−) side. Accordingly, the right ear in the Right-CS group and the left ear in the Left-CS group were pooled as the CS(+) side, whereas the contralateral ears were pooled as the CS(−) side.

For caloric analysis, the ear-specific caloric response was quantified as the sum of the peak SPVs elicited by warm and cool irrigations in each ear (e.g., right ear response = right warm peak SPV + right cool peak SPV). The canal paresis (CP) value was calculated using Jongkees’ formula, and both SPV and CP values were compared between groups to investigate the relationship between the side of CS and caloric asymmetry. A CP value greater than 25% was generally considered abnormal in clinical practice; however, CP was analyzed as a continuous variable rather than being dichotomized as normal or abnormal in the present study. Patients with bilateral vestibular hypofunction, defined as a total peak slow-phase velocity (SPV) of <6°/s on both sides in the caloric test [12], were excluded only from the CP analysis to avoid overestimation of CP values with limited clinical relevance. These subjects remained eligible for all other analyses. Because caloric testing evaluates horizontal canal function, all comparisons between caloric parameters and CS were restricted to horizontal canal vHIT findings.

### 2.5. Main Data Analysis

For each subject, the mean VOR gain and caloric SPV were compared between the CS(+) and CS(−) sides within the unilateral-CS group. Side-specific analyses were also performed separately for the Right-CS and Left-CS subgroups. Canal paresis values were compared among the Right-CS, Left-CS, and Bilateral-CS groups. Additionally, demographic characteristics (age and sex) were analyzed across the three groups.

### 2.6. Statistical Analysis

Statistical analyses were performed using SPSS software (version 22.0, IBM Corp., Armonk, NY, USA). Normality was assessed using the Shapiro–Wilk test and visual inspection of Q–Q plots. Given the large sample size, parametric tests were considered appropriate for the analyses. One-way analysis of variance (ANOVA) followed by Tukey’s post hoc test was employed to compare age and canal paresis among the three CS groups. Paired *t*-tests were performed to compare the VOR gain and caloric SPV between the CS(+) and CS(−) sides within each group. The asymmetry in the occurrence of CS between the right and left sides was assessed using McNemar’s test. A *p*-value less than 0.05 was considered to indicate statistical significance.

## 3. Results

### 3.1. Distribution and Lateral Asymmetry of Corrective Saccades

A total of 1573 subjects underwent vestibular function testing (mean age, 55.7 ± 17.6 years; 582 males and 991 females). Among them, 1174 subjects (74.6%) with normal horizontal VOR gains on both sides were included in the CS analysis. Within this subgroup, 169 (14.4%) showed CS only on the right side (Right-CS group), 97 (8.3%) only on the left side (Left-CS group), 278 (23.7%) on both sides (Bilateral-CS group), and 630 (53.7%) on neither side. McNemar’s test revealed a significant right–left asymmetry, with CS occurring more frequently on the right side (χ^2^ = 18.95, *p* < 0.0001) (Table 1).

### 3.2. Demographic Characteristics of the Groups

The mean age differed significantly among the three groups (F(2, 541) = 14.29, *p* < 0.001, η_p_^2^ = 0.050, 95% CI 0.019–0.088). Post hoc analysis using Tukey’s HSD test revealed that the Bilateral-CS group (63.6 ± 14.4 years) was significantly older than the other two groups, Right-CS (57.4 ± 15.9 years, *p* < 0.001) and Left-CS (55.6 ± 16.4 years, *p* < 0.001). However, there was no significant difference in age between the Right-CS and Left-CS groups (*p* = 0.63) (Table 1). Sex distribution did not differ significantly among the three groups (χ^2^ = 2.46, *p* = 0.292) (Table 1).

When analyzed separately by sex, a similar trend was observed. Among male subjects, mean age differed significantly among the three groups (F(2, 204) = 6.13, *p* = 0.003, η_p_^2^ = 0.057, 95% CI 0.008–0.122), with the Bilateral-CS group (63.3 ± 15.0 years) being older than both the Right-CS group (56.7 ± 17.8 years, *p* = 0.036) and the Left-CS group (53.3 ± 18.8 years, *p* = 0.005). No significant difference was observed between the Right-CS and Left-CS groups (*p* = 0.57). Similarly, among female subjects, mean age differed significantly among the groups (F(2, 334) = 7.84, *p* < 0.001, η_p_^2^ = 0.045, 95% CI 0.009–0.092), with the Bilateral-CS group (63.9 ± 14.1 years) being older than both the Right-CS group (57.9 ± 14.5 years, *p* = 0.003) and the Left-CS group (57.3 ± 14.4 years, *p* = 0.008), whereas no significant difference was found between the Right-CS and Left-CS groups (*p* = 0.97) (Table 1).

### 3.3. Comparison of VOR Gain and Caloric Response in the Unilateral-CS Group

**Figure 1:**
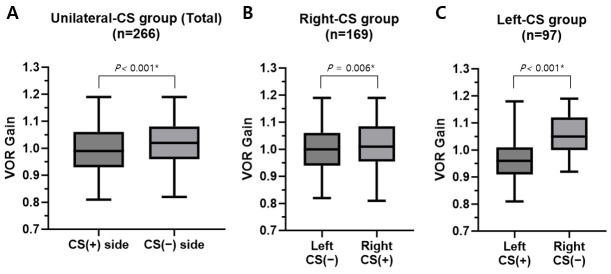
VOR gain asymmetry between CS(+) and CS(−) sides in the unilateral-CS group and subgroups by CS side. VOR, vestibulo-ocular reflex; CS, corrective saccades. Boxes represent the interquartile range (IQR), and horizontal lines indicate medians. * All *p*-values were determined using paired *t*-test. For unilateral-CS subjects (*n* = 266), the side exhibiting CS was designated as the CS(+) side and the contralateral side without CS as the CS(−) side. (**A**) Comparison of VOR gains between the CS(+) and CS(−) sides in all unilateral-CS patients (*n* = 266). (**B**) Right-CS subgroup (*n* = 169): VOR gain was slightly but significantly higher on the CS(+) side, indicating right-sided gain dominance. (**C**) Left-CS subgroup (*n* = 97): VOR gain was significantly lower on the CS(+) side compared with the CS(−) side. While the overall analysis (**A**) showed lower VOR gains on the CS(+) side, the Right-CS subgroup (**B**) demonstrated a reversed pattern, suggesting inherent rightward gain asymmetry.

**Figure 2:**
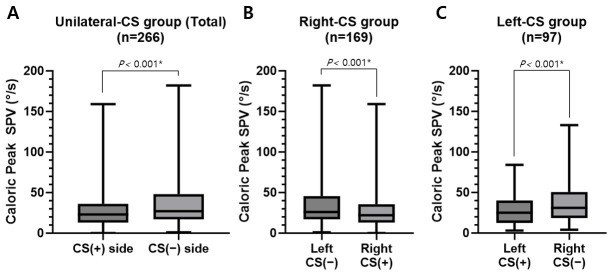
Caloric response differences between CS(+) and CS(−) sides in the unilateral-CS group and subgroups by CS side. CS, corrective saccades; SPV, slow-phase velocity. * All *p*-values were determined using paired *t*-test. For unilateral-CS subjects (*n* = 266), the side exhibiting CS was designated as the CS(+) side and the contralateral side without CS as the CS(−) side. (**A**) Comparison of caloric peak SPV between the CS(+) and CS(−) sides in all unilateral-CS patients (*n* = 266). (**B**) Comparison between the left and right sides in the Right-CS subgroup (*n* = 169). (**C**) Comparison between the left and right sides in the Left-CS subgroup (*n* = 97). In all analyses, the caloric response was significantly lower on the CS(+) side than on the CS(−) side (*p* < 0.001), indicating reduced caloric response on the side showing CS.

In the total unilateral-CS group, the mean VOR gain was significantly lower on the CS(+) side than on the CS(−) side (1.00 ± 0.09 vs. 1.02 ± 0.09, mean difference, −0.024; 95% CI, −0.035 to −0.012; *p* < 0.001). Similarly, the caloric response showed a significantly lower peak SPV on the CS(+) side compared with the CS(−) side (28.18 ± 22.71°/s vs. 35.73 ± 27.58°/s, mean difference, −7.55°/s; 95% CI, −9.98 to −5.11°/s; *p* < 0.001) (Table 2, Figure 1A and Figure 2A).

### 3.4. Side-Specific Analysis of the Right-CS and Left-CS Groups

When analyzed separately according to the side of the CS, the Right-CS group showed a slightly but significantly higher VOR gain on the right side than on the left side (1.02 ± 0.09 vs. 1.00 ± 0.09, mean difference, 0.018; 95% CI, 0.005 to 0.031; *p* = 0.006), which was an unexpected finding given the lower caloric response on the same side (28.07 ± 24.62°/s vs. 35.31 ± 29.91°/s, mean difference, −7.24°/s; 95% CI, −10.51 to −3.98°/s; *p* < 0.001). In contrast, the Left-CS group showed a significantly lower VOR gain on the left side than on the right side (0.96 ± 0.08 vs. 1.06 ± 0.07, mean difference, −0.096; 95% CI, −0.111 to −0.081; *p* < 0.001), with a corresponding reduction in the caloric response on the left side (28.40 ± 19.07°/s vs. 36.46 ± 23.10°/s, mean difference, −8.08°/s; 95% CI, −11.66 to −4.51°/s; *p* < 0.001) (Table 2, Figure 1B,C and Figure 2B,C).

Overall, caloric responses were consistently reduced on the CS(+) side, supporting an association between CS and lateralized vestibular asymmetry. In contrast, VOR gain differences were not uniformly aligned with this pattern, particularly in the Right-CS group, where a slightly higher gain was observed despite lower caloric responses.

### 3.5. Canal Paresis Differences Among the Groups

**Figure 3:**
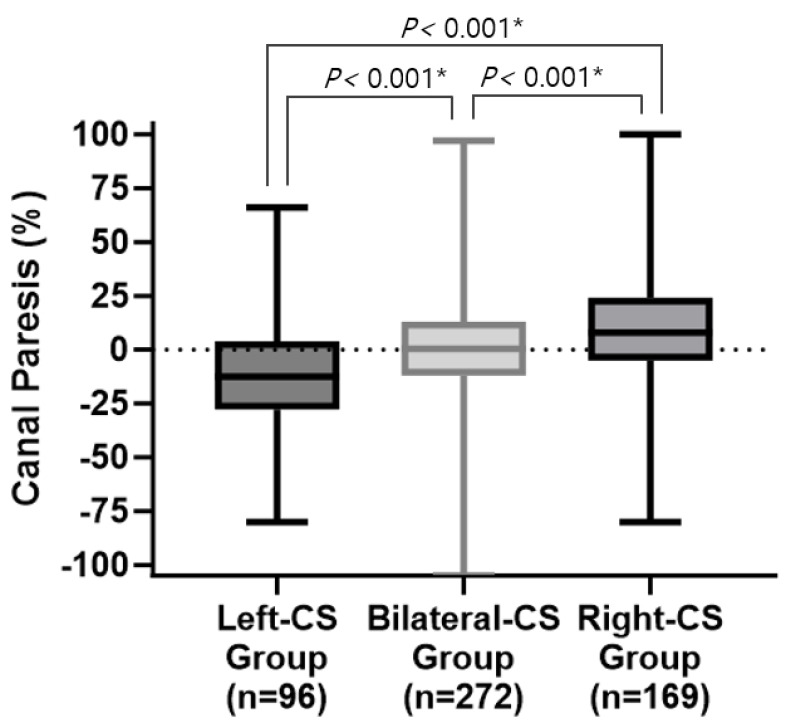
Comparison of canal paresis values among the Left-CS, Bilateral-CS, and Right-CS groups. CS, corrective saccades. * All *p*-values were determined using one-way ANOVA followed by Tukey’s post hoc test. The Right-CS group exhibited significantly higher CP values, and the Left-CS group significantly lower CP values, indicating opposite lateralization of canal weakness.

The mean canal paresis (CP) value differed significantly among the three groups (F(2, 534) = 24.22, *p* < 0.001, η_p_^2^ = 0.083, 95% CI 0.042–0.129). Post hoc Tukey’s test revealed that the Right-CS group (11.41 ± 28.8%) had significantly higher CP values than Bilateral-CS (0.03 ± 29.6%, *p* < 0.001) and the Left-CS (−13.98 ± 26.3%, *p* < 0.001) groups. In addition, the Left-CS group showed significantly lower CP values than the Bilateral-CS group (*p* < 0.001) (Table 1, Figure 3). Positive CP values in the Right-CS group indicated right-sided canal weakness, whereas negative CP values in the Left-CS group indicated left-sided canal weakness. These findings indicate that the side of caloric weakness corresponded to the side exhibiting CS.

Taken together, these findings suggest that the side showing CS on vHIT tends to correspond to the side of reduced caloric response, even when vHIT gains remain within the normal range.

## 4. Discussion

### 4.1. Summary of Key Findings

In this study, we observed that the pattern of CS despite normal vHIT gains is not uncommon in clinical practice. The side exhibiting CS frequently corresponded to the side with reduced caloric response, suggesting that CS may be associated with subtle unilateral vestibular hypofunction that is not reflected by reduced VOR gain [7]. In addition, patients with bilateral CS were significantly older than those with unilateral CS, suggesting that age-related effects on vestibular function or central compensation mechanisms may contribute to this pattern [13,14]. Furthermore, the predominance of right-sided CS and the trend toward higher right-side VOR gain suggest that factors such as methodological asymmetry or biological lateralization may contribute to this directional tendency.

### 4.2. Interpretation of Normal VOR Gain with Corrective Saccades

Although normal VOR gain and CS may appear contradictory, they are not mutually exclusive by definition. The clinical cut-off for abnormal gain is typically set at 0.8 for the horizontal semicircular canals [5,13,15,16,17,18,19], whereas the definition of CS varies among studies—often based on the number or proportion (%), velocity, or consistency of refixation saccades detected during repeated impulses. Because the two conditions are defined independently, a subset of cases may satisfy both criteria, and the observed proportion of such cases is therefore expected to vary depending on how each criterion is operationally defined.

Notably, Anson et al. (2016) reported that even within the normal range, subjects with VOR gain between 0.8 and 0.9 exhibited more frequent and larger CS compared to those with gain between 0.9 and 1.0, suggesting that subtle vestibular deficits may manifest as CS before gain reduction as defined by the clinical threshold [14]. In a related study by the same authors [14], regression analyses adjusted for age, sex, and race demonstrated that the percentage of HITs with compensatory saccades increased by 4.5% for every 0.1 decrease in VOR gain (*p* < 0.0001). In contrast, overt saccade amplitude decreased by 0.6° (*p* < 0.005) and latency increased by 90 ms (*p* < 0.001) for every 0.1 increase in gain. These findings indicate that the relationship between gain and saccadic behavior is better understood as a continuum rather than a dichotomy, in which relatively small variations in gain may already elicit compensatory eye movements.

Taken together, these observations suggest that the apparent coexistence of normal VOR gain and CS largely reflects the definitional nature of this issue, rather than a true categorical contradiction. Accordingly, adjusting the gain threshold or modifying CS criteria—even slightly—would alter the proportion of patients classified as having normal VOR gain with CS.

Differences in study populations may also influence the prevalence of CS despite preserved VOR gain. Although we applied criteria similar to those of Yang et al. [5], our cohort consisted of symptomatic patients who underwent vestibular testing rather than healthy individuals, which may partly explain the higher prevalence of normal-gain CS observed in our study.

### 4.3. Association Between Corrective Saccades and Caloric Asymmetry

In the present study, the side showing CS on vHIT corresponded to the side with reduced caloric response. This observation is consistent with the hypothesis of subthreshold unilateral vestibular hypofunction, despite preserved VOR gain.

At the same time, this finding may be interpreted in several non-exclusive ways. Given the differential sensitivities of vestibular tests, low-frequency canal function assessed by caloric stimulation may decline earlier, while high-frequency VOR gain remains relatively preserved [20]. In this context, mild caloric asymmetry with subtle CS may indicate an early manifestation of vestibular dysfunction. Alternatively, high-frequency VOR gain may normalize before low-frequency vestibular function recovers, with residual CS or caloric weakness indicating partial adaptation or incomplete recovery [21].

Taken together, the observation of reduced caloric response in patients with CS despite preserved VOR gain is consistent with a mild imbalance in vestibular function along a continuous spectrum, rather than a coincidental or contradictory finding.

### 4.4. Bilateral Corrective Saccades and Age-Related Effects

Subjects in the bilateral-CS group were significantly older than those in either unilateral-CS group, whereas the two unilateral groups did not differ in age. This finding should be interpreted cautiously. Rather than demonstrating that bilateral CS become more frequent with advancing age, our cross-sectional data indicate that the subgroup characterized by bilateral CS was, on average, older than the subgroups characterized by unilateral CS.

One possible explanation is that bilateral CS reflect a relatively symmetric decline in vestibular function associated with aging. Age-related reductions in vestibular hair cells and afferent responsiveness affect both labyrinths and may produce subtle bilateral vestibular deficits before a clinically abnormal reduction in VOR gain becomes apparent [22]. In contrast, unilateral CS in our cohort were associated with caloric asymmetry and likely reflected lateralized vestibular hypofunction. Thus, bilateral CS may represent a more diffuse, age-related process rather than a focal vestibular deficit.

However, the association between bilateral CS and age was observed despite restricting the analysis to subjects with normal bilateral VOR gain. Although Anson et al. examined compensatory saccade amplitude rather than CS laterality [14,22], their findings may help explain our observations. They demonstrated that compensatory saccade amplitude increased with age even after controlling for VOR gain and proposed that this phenomenon may reflect both subtle vestibular decline and age-related alterations in central gaze-stabilization mechanisms. These processes may also contribute to the emergence of bilateral CS in older individuals despite preserved VOR gain.

The functional significance of age-associated bilateral CS remains uncertain. Although compensatory saccades are generally regarded as an adaptive mechanism that helps maintain gaze stability, they may also reflect incomplete compensation or age-related deterioration of central gaze-stabilization mechanisms. If so, bilateral CS could represent not only a compensatory response to subtle vestibular decline but also a marker of residual gaze instability. Larger compensatory saccades have been hypothesized to contribute to oscillopsia and reduced dynamic visual stability during walking, potentially affecting functional mobility in older adults [22]. Because our data are cross-sectional, we cannot determine whether bilateral CS in older subjects represent successful adaptation or a maladaptive response. Therefore, caution is warranted when inferring real-world gaze stabilization or mobility from the presence of CS alone.

In addition, alternative explanations should also be considered, including increased noise or artifacts, reduced fixation stability, comorbid ocular or neurological conditions, and examiner-related factors such as goggle fit, all of which may be more prevalent in older adults.

### 4.5. Right-Sided Predominance: Methodological and Functional Considerations

The predominance of right-sided CS and slightly higher right-side VOR gains observed in this study may arise from both methodological and physiological factors. From a technical standpoint, most examiners are right-handed, which can lead to stronger or more stable rightward impulses, exposing subtle VOR deficits more readily on that side [23].

In addition, most commercial vHIT systems record only the right eye, which may introduce asymmetry in both gain measurement and CS detection. Camera alignment, calibration errors, and goggle slippage can disproportionately affect responses recorded from a single eye [18]. Monocular recording is also associated with a well-recognized adduction–abduction bias, whereby adducting eye movements during rightward impulses tend to produce slightly higher gain values than abducting eye movements during leftward impulses. This phenomenon has been demonstrated in both methodological and normative studies and may partly explain the tendency toward higher right-sided gains observed in our cohort [10,24,25,26,27,28].

At the participant level, asymmetries may also exist in gaze fixation ability and compensatory eye-movement strategies between the two sides. A biological contribution cannot be excluded, as previous studies have suggested possible lateral asymmetries within vestibulo-ocular pathways [27]. However, evidence supporting clinically meaningful right–left differences in normal vestibular function remains limited. In addition, slightly elevated vHIT gains have occasionally been reported in certain central vestibular disorders [29]. Because central vestibular pathology was not systematically evaluated in the present study, the potential contribution of central mechanisms cannot be completely excluded. Overall, technical factors—including examiner handedness, monocular right-eye recording, and the associated adduction–abduction bias—are likely to account for at least part of the lateralized findings observed in the present study.

Although the right-sided gain was statistically higher in the Right-CS group, the absolute difference was very small (mean difference, 0.02) and is unlikely to be clinically meaningful. Rather than indicating superior vestibular function on the CS side, this finding may reflect the coexistence of two competing effects: a tendency for the CS side to show slightly lower VOR gain and a modest rightward gain asymmetry observed throughout the cohort. In the Right-CS group, the latter effect may have marginally outweighed the former, resulting in a statistically significant but quantitatively negligible increase in right-sided gain.

Taken together, these considerations underscore the importance of balanced testing protocols and careful interpretation of lateralized CS patterns, particularly when subtle gain differences are present.

### 4.6. Definition and Detection of Corrective Saccades

There is currently no universally accepted definition for what constitutes a true CS. In Yang et al. [5], the presence of CS was defined when saccades appeared in more than two of ten impulses, without restriction on the peak velocity of saccades. Kabaya et al. [6] proposed a stricter definition, requiring saccades with a peak velocity exceeding 100°/s in more than 50% of impulses. In addition to frequency and peak velocity criteria, some studies have restricted the temporal window within which a saccade must occur to be classified as a CS. For example, Anson et al. restricted analysis to saccades occurring between 25 and 503 ms after head impulse onset to exclude volitional saccades and ensure that only reflexive, vestibular-driven responses were analyzed [22]. These varying definitions reflect ongoing efforts to distinguish physiologically meaningful corrective responses from artifacts, spontaneous eye movements, and measurement noise.

However, the definition of CS is inherently influenced by threshold selection. Stricter criteria based on frequency, consistency, velocity, or latency may improve specificity, but they may also exclude subtle corrective responses associated with mild vestibular dysfunction. Similar to VOR gain, which is often dichotomized using arbitrary clinical cut-offs, the classification of CS as present or absent may depend heavily on where a threshold is placed. Such dichotomization may oversimplify what is fundamentally a continuous compensatory process.

In the present study, we adopted a relatively conservative but inclusive approach, combining automated detection with visual verification and defining CS as the presence of at least three reproducible CS on one side. Importantly, we did not impose a strict peak-velocity threshold, allowing detection of relatively mild but consistent corrective responses. Although such saccades may occasionally be dismissed as artifacts or normal variants, our analysis identified clinically relevant associations, including the correspondence between the side exhibiting CS and the side showing weaker caloric responses, even under less stringent CS criteria. This suggests that even relatively mild CS may carry clinical relevance and should not be dismissed solely because they fail to meet more stringent velocity or frequency criteria.

Future efforts should focus not only on establishing standardized quantitative criteria for CS, but also on identifying which CS characteristics—such as frequency, amplitude, velocity, consistency, or latency—most accurately reflect clinically meaningful vestibular dysfunction. Recognition of the continuous nature of compensatory eye movements will be essential for developing a more clinically relevant framework for CS interpretation.

### 4.7. Clinical Implications

From a clinical standpoint, the coexistence of normal VOR gain and CS should not be dismissed as a benign variant or measurement artifact [30]. Our findings suggest that gain alone may not fully capture vestibular dysfunction and that clinically relevant abnormalities may be present despite preserved VOR gain. Therefore, the presence of CS should be considered an important component of vHIT interpretation and evaluated in conjunction with clinical findings and complementary vestibular tests [30,31]. Furthermore, because lateral asymmetries in vHIT measurements have been reported previously, lateralized CS findings should be interpreted with awareness of potential methodological influences, including examiner technique and monocular eye recording.

### 4.8. Limitations and Future Directions

Several limitations should be acknowledged. First, subgroup analyses according to specific vestibular diagnoses were not performed because the primary aim of this study was to characterize vestibular test findings associated with CS despite normal VOR gain and to explore their clinical interpretation, rather than investigate disease-specific mechanisms. Furthermore, reliable disease-specific classification was not feasible in this retrospective cohort because of heterogeneous disease stages and incomplete diagnostic information. Future studies involving well-defined diagnostic groups may provide additional insight into disease-specific mechanisms and CS patterns.

Second, we did not differentiate covert and overt CS, focusing instead on the presence and laterality of CS. Future studies examining the temporal characteristics of CS may further clarify the mechanisms underlying compensatory eye movements.

Third, directional preponderance (DP) was not analyzed in the present study. Although canal paresis was selected as the primary caloric parameter because it is widely used to assess lateralized vestibular weakness, future studies incorporating DP analysis may provide additional insight into caloric asymmetry associated with CS.

Finally, the cross-sectional design precludes conclusions regarding the temporal and functional significance of CS. Whether CS represent early compensation, progressive vestibular decline, successful adaptation, or maladaptive responses remains uncertain. Longitudinal studies and further standardization of CS metrics will be necessary to clarify their clinical significance.

## 5. Conclusions

CS were frequently observed in subjects with normal VOR gain and were associated with weaker caloric responses on the corresponding side. In addition, bilateral CS were associated with older age, suggesting that age-related changes in vestibular and compensatory gaze-stabilization mechanisms may contribute to their occurrence even when VOR gain remains within the normal range. These findings suggest that CS observed despite normal VOR gain should not be dismissed as a benign or meaningless finding and may represent a subtle marker of vestibular dysfunction or asymmetry that is not fully captured by gain measurements alone.

The present results highlight the importance of incorporating CS into routine vHIT interpretation and support a more integrated approach that combines gain measurements, CS characteristics, complementary vestibular tests, and overall clinical context. Future studies should further refine the diagnostic utility of normal-gain CS through standardized definitions, quantitative criteria, and multicenter validation.

## Figures and Tables

**Table 1:** Demographic characteristics and canal paresis in the Unilateral-CS (Right and Left) and Bilateral-CS groups.

Variable	Unilateral-CS Group		Bilateral-CS Group	Statistics	*p*-Value	Bilateral Normal Gain	Overall Cohort
	Right-CS Group	Left-CS Group					
**Number of subjects,** ***n*** (**%**)	169 (14.4%)	97 (8.3%)	278 (23.7%)	N/A	<0.001 *	1174 (100%)	1573
Age (years)	57.4 ± 15.9	55.6 ± 16.4	63.6 ± 14.4	F(2, 541) = 14.29 η_p_^2^ = 0.050	<0.001 *	53.3 ± 18.8	55.7 ± 17.6
**Male,** ***n*** (**%**)	69 (40.8%)	41 (42.3%)	97 (34.9%)			406 (34.6%)	582 (37.0%)
Age (years)	56.7 ± 17.8	53.3 ± 18.8	63.3 ± 15.0	F(2, 204) = 6.13 η_p_^2^ = 0.057	0.003 *	52.6 ± 18.7	54.3 ± 18.6
**Female,** ***n*** (**%**)	100 (59.2%)	56 (57.7%)	181 (65.1%)			768 (65.4%)	991 (63.0%)
Age (years)	57.9 ± 14.5	57.3 ± 14.4	63.9 ± 14.1	F(2, 334) = 7.84 η_p_^2^ = 0.045	<0.001 *	55.2 ± 17.0	56.5 ± 16.9
**CP (%)**	11.41 ± 28.8	−13.98 ± 26.3	0.03 ± 29.6	F(2, 534) = 24.22 η_p_^2^ = 0.083	<0.001 *		

CS, corrective saccades; CP, canal paresis. Values are presented as mean ± SD or *n* (%). Percentages for the number of subjects are based on the total normal-gain cohort (*n* = 1174), whereas percentages for male and female subjects are calculated within each CS group. The *p*-value for the distribution of subjects was determined using McNemar’s test. Age and CP comparisons were performed using one-way ANOVA. * Indicates statistical significance (*p* < 0.05). Cases with bilateral vestibular hypofunction (total slow-phase velocity < 6°/s on both sides) were excluded from the canal paresis analysis to avoid overestimated CP values with limited clinical relevance.

**Table 2:** Comparison of vestibular function parameters in the unilateral-CS group and subgroups by CS side.

Groups	VOR Gain CS(+) Side	VOR Gain CS(−) Side	*p*-Value	Caloric Peak SPV CS(+) Side (°/s)	Caloric Peak SPV CS(−) Side (°/s)	*p*-Value
**Unilateral-CS Group** (**Total**) (*n* = 266)	1.00 ± 0.09	1.02 ± 0.09	<0.001 *	28.18 ± 22.71	35.73 ± 27.58	<0.001 *
	**VOR Gain** (**Left**)	**VOR Gain** (**Right**)	***p*** **-Value**	**Caloric Peak SPV** (**Left**) (**°/s**)	**Caloric Peak SPV** (**Right**) (**°/s**)	***p*** **-Value**
**Right-CS Group** (*n* = 169)	**1.00 ± 0.09**	**1.02 ± 0.09**	0.006 *	35.31 ± 29.91	28.07 ± 24.62	<0.001 *
**Left-CS Group** (*n* = 97)	0.96 ± 0.08	1.06 ± 0.07	<0.001 *	28.40 ± 19.07	36.46 ± 23.10	<0.001 *

VOR, vestibulo-ocular reflex; CS, corrective saccades; SPV, slow-phase velocity. * All *p*-values were determined using paired *t*-test.

## Data Availability

The original contributions presented in this study are included in the article. Further inquiries can be directed to the corresponding author.
